# Novel Tryptophan Metabolism by a Potential Gene Cluster That Is Widely Distributed among Actinomycetes*

**DOI:** 10.1074/jbc.M112.436451

**Published:** 2013-02-19

**Authors:** Taro Ozaki, Makoto Nishiyama, Tomohisa Kuzuyama

**Affiliations:** From the Biotechnology Research Center, the University of Tokyo, 1-1-1 Yayoi, Bunkyo-ku, Tokyo 113-8657, Japan

**Keywords:** Biosynthesis, Enzymes, Flavin, Isoprenoid, Metabolism, Tryptophan, *Streptomyces*

## Abstract

The characterization of potential gene clusters is a promising strategy for the identification of novel natural products and the expansion of structural diversity. However, there are often difficulties in identifying potential metabolites because their biosynthetic genes are either silenced or expressed only at a low level. Here, we report the identification of a novel metabolite that is synthesized by a potential gene cluster containing an indole prenyltransferase gene (*SCO7467*) and a flavin-dependent monooxygenase (FMO) gene (*SCO7468*), which were mined from the genome of *Streptomyces coelicolor* A3(2). We introduced these two genes into the closely related *Streptomyces lividans* TK23 and analyzed the culture broths of the transformants. This process allowed us to identify a novel metabolite, 5-dimethylallylindole-3-acetonitrile (5-DMAIAN) that was overproduced in the transformant. Biochemical characterization of the recombinant SCO7467 and SCO7468 demonstrated the novel l-tryptophan metabolism leading to 5-DMAIAN. SCO7467 catalyzes the prenylation of l-tryptophan to form 5-dimethylallyl-l-tryptophan (5-DMAT). This enzyme is the first actinomycetes prenyltransferase known to catalyze the addition of a dimethylallyl group to the C-5 of tryptophan. SCO7468 then catalyzes the conversion of 5-DMAT into 5-dimethylallylindole-3-acetaldoxime (5-DMAIAOx). An aldoxime-forming reaction catalyzed by the FMO enzyme was also identified for the first time in this study. Finally, dehydration of 5-DMAIAOx presumably occurs to yield 5-DMAIAN. This study provides insight into the biosynthesis of prenylated indoles that have been purified from actinomycetes.

## Teaser

**Background:** Potential gene clusters involving indole prenyltransferase are widely distributed in actinomycetes.

**Results:** One gene cluster was found to be responsible for 5-dimethylallylindole-3-acetonitrile biosynthesis.

**Conclusion:** The biosynthetic route leading to 5-dimethylallylindole-3-acetonitrile represents a novel tryptophan metabolism.

**Significance:** This result provides insight into the biosynthesis of prenylated indole derivatives that have been purified from actinomycetes.

## Introduction

Natural products have been an important resource for drug discovery and development over the last 3 decades. Actinomycetes are a rich source of natural products, and a wide variety of these chemicals have been used as medicinal drugs (1, 2). Recently, however, the screening of bioactive compounds from microorganisms has often resulted in the identification of known compounds. The decreasing hit rate for identifying new compounds has reduced the advantages of screening natural products. However, genome sequencing of actinomycetes has highlighted numerous potential areas with metabolic diversity and revealed the existence of a number of potential gene clusters with unknown function (3–5). In fact, the number of potential gene clusters is significantly more than the number of metabolites observed under standard fermentation conditions employed in laboratories (6).

To uncover the function of potential gene clusters that might code for the biosynthesis of secondary metabolites, genome sequence-guided identification of metabolites has been performed in combination with heterologous expression, gene knock-out and complementation analyses, and silent gene activation studies (7, 8). Normally, it is difficult to elucidate the function of a potential gene cluster and to identify the metabolite(s) associated with the cluster unless an appropriate system for heterologous expression is available (9), the gene clusters are highly expressed under normal conditions (8), or a pathway-specific regulator is identified (7). In this study, we characterized a gene cluster by employing a relatively simple strategy that is based on a gene dosage effect. We hypothesized that this introduction of some structural genes from a targeted gene cluster into a heterologous host using a plasmid vector would allow us to detect the production of a targeted product that has never been detected.

**FIGURE 1.**
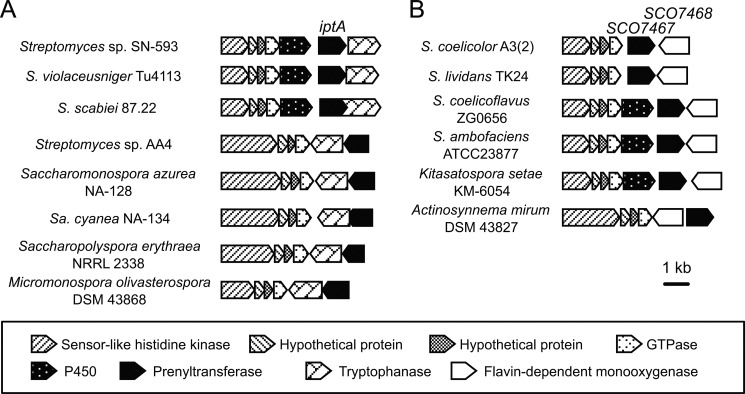
**Gene clusters containing the indole prenyltransferase gene.** The Type A gene clusters contain tryptophanase genes. This type of cluster is associated with the biosynthesis of 6-dimethylallylindole-3-carbardehyde. The Type B gene clusters contain flavin-dependent monooxygenase genes. The function of the Type B clusters has not been identified.

We focused on a selected group of putative gene clusters including an indole prenyltransferase IptA homolog because it is widely distributed among actinomycetes (Fig. 1). We classified the gene clusters into two types on the basis of their constituent genes; in addition to the indole prenyltransferase gene, one gene cluster contained a tryptophanase gene, whereas the other contained a flavin-dependent monooxygenase (FMO)^3^ gene (Fig. 1). Takahashi *et al.* (10) have demonstrated that the former type of gene cluster in *Streptomyces* sp. SN-593 is responsible for the biosynthesis of 6-dimethylallylindole (DMAI)-3-carbardehyde. In this biosynthetic pathway, IptA catalyzes the addition of a dimethylallyl group onto the C-6 position of tryptophan to yield 6-dimethylallyltryptophan (DMAT). No metabolic pathways involving the latter type of gene clusters have yet been elucidated, although FMO gene clusters are also widely distributed among actinomycetes. Here, we report the identification of a novel metabolite that is synthesized by the *iptA* homolog- and FMO-containing gene cluster in *Streptomyces coelicolor* A3(2), the genome sequence of which was completed >10 years ago. We introduced the *SCO7467* gene and the FMO gene *SCO7468*, which form a gene cluster in the *S. coelicolor* A3(2) genome, into the heterologous host *Streptomyces lividans* TK23, which possesses a gene cluster almost identical to that of *S. coelicolor* A3(2). The introduction of these two genes enabled us to identify a novel metabolite, 5-DMAI-3-acetonitrile (5-DMAIAN), in the *S. lividans* transformant. In addition, we elucidated the biochemical functions of SCO7467 and SCO7468 in the biosynthesis of 5-DMAIAN and identified the presence of a novel tryptophan metabolism. The elucidation of this novel tryptophan metabolism provides insight into the biosynthesis of prenylated indole derivatives that have been purified from actinomycetes.

## EXPERIMENTAL PROCEDURES

### 

#### 

##### Reagents, Bacterial Strains, Plasmids, and Culture Conditions

Tryptophan, indole-3-pyruvic acid, NADPH, NADH, FAD, and FMN were purchased from Sigma-Aldrich. Indole-3-acetonitrile (IAN) and hydroxylammonium chloride were purchased from Tokyo Chemical Industry. 2-Picoline-borane was purchased from Junsei Chemical (Tokyo, Japan). A mixture of *syn*- and *anti*-isomers of indole-3-acetaldoxime (IAOx) was a gift from Dr. Hiroyuki Kasahara (RIKEN, Yokohama, Japan). The bacterial strains, plasmids, and primers used in this study are listed in Table 1. The *Streptomyces-Escherichia coli* shuttle vector pSE101 (11) was used for the heterologous expression of genes in *S. lividans* TK23. The genes cloned downstream of the *lac* promoter in pSE101 are efficiently expressed in *S. lividans* TK23 (12). The transformants of *S. lividans* TK23 cultures were routinely grown at 30 °C on TSB agar plates (3% tryptone soya broth (Oxoid, England), 1.5% agar). For the analysis of metabolites produced by the transformants, a scrape of the mycelium on the culture plate was inoculated into a test tube containing 10 ml of TSB tsr medium (3% tryptone soya broth and 30 μg ml^−1^ thiostrepton) for 2 days at 30 °C on a reciprocal shaker at 300 rpm. Two milliliters of the preculture was inoculated into 500-ml baffled flasks containing 100 ml of TSB tsr medium, and the culture was incubated for 4 days at 27 °C on a rotary shaker at 160 rpm. *Escherichia coli* was grown at 37 °C in LB broth or Terrific broth (13). Vector pHIS8 (14) was used for the heterologous expression of genes in *E. coli* BL21(DE3). Ampicillin (100 μg ml^−1^), or kanamycin (50 μg ml^−1^) was added for the selection of plasmid-containing *E. coli* cells.

**TABLE 1:**
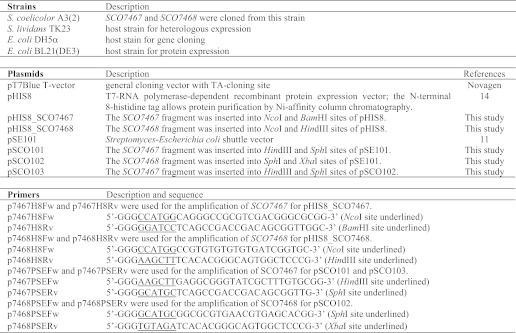
**Strains, plasmids, and primers used in this study**

##### Construction of Heterologous Expression Plasmids

The *SCO7467* and *SCO7468* genes were amplified from genomic DNA of *S. coelicolor* A3(2), using the Expand High Fidelity PCR system (Roche Diagnostics Japan, Tokyo, Japan) and the primers listed in Table 1. Primers were designed to amplify each gene from the upstream region that included a ribosomal binding site. A PCR fragment containing the *SCO7468* gene was cloned downstream of the *lac* promoter in pSE101 to give pSCO102. Similarly, a PCR fragment containing the *SCO7467* gene was cloned into pSE101 to give pSCO101 and into pSCO102 to give pSCO103. Each plasmid was introduced into *S. lividans* TK23 by a polyethylene glycol-mediated protoplast method (15).

##### LC-MS/MS Analysis of the Metabolite Produced by S. lividans TK23 Transformants

The culture broths of the transformants were extracted twice with ethyl acetate. The ethyl acetate fraction was concentrated *in vacuo* and then dissolved in methanol. The resultant methanol solution was analyzed on an LC-MS/MS system equipped with a CAPCELL PAK C_18_ IF column (2.0 × 50 mm; column temperature, 40 °C; Shiseido, Tokyo, Japan) under the following conditions: mobile phase A, water + 0.1% formate; mobile phase B, acetonitrile + 0.1% formate; 10–90% B over 4 min, 90% B for 1 min, and then 10% A for 5 min, at a flow rate of 0.4 ml min^−1^. A high resolution MS apparatus (AB Sciex Triple TOF^TM^ 5600, Tokyo, Japan) was used to monitor metabolites. The MS and MS/MS analyses were simultaneously performed using electrospray ionization in the positive ion mode. The enzymatic reaction with the recombinant SCO7467 and SCO7468 was analyzed under the same conditions. If necessary, an SPD-M20A photodiode array (Shimadzu, Kyoto, Japan) was also used.

##### Isolation and Structural Determination of 5-DMAIAN

Two liters of culture broth of the transformant was extracted with an equal volume of acetone and then concentrated to remove the solvent. The remaining water fraction was extracted twice with an equal volume of ethyl acetate, and the organic layer was then concentrated *in vacuo*. The remaining residue was resolved in methanol and passed through Sep-Pak Plus C18 Cartridges (Waters, Ireland). The eluted fraction was again concentrated *in vacuo* to give 300 mg of a residue. The concentrated extract was separated by preparative HPLC on a Senshu Pak PEGASIL ODS column (20 × 250 mm, Senshu Scientific, Tokyo, Japan) and an isocratic elution of 75% methanol at a flow rate of 8 ml min^−1^. The column eluate was monitored at 271 nm. Finally, 8.9 mg of 5-DMAIAN was obtained.

##### Structural Analysis

The structures of the unidentified products were analyzed using ^1^H NMR spectroscopy and ^13^C NMR spectroscopy (600 MHz, JEOL ECA-600; Tokyo, Japan). The high resolution MS apparatus was used to determine the molecular formulas of the reaction products. The MS analysis was performed using electrospray ionization in the positive-ion mode.

##### NMR and MS Spectral Data of 5-DMAIAN

^1^H NMR (DMSO-*d6*) δ: 1.68 (s, 3H, H-5′), 1.69 (s, 3H, H-4′), 3.36 (d, *J*_H_ = 6.9 Hz, 2H, H-1′), 3.97 (s, 2H, H-8), 5.31 (t, *J*_H_ = 6.9 Hz, 1H, H-2′), 6.93 (dd, *J*_H_ = 1.4, 8.2 Hz, 1H, H-6), 7.26 (d, *J*_H_ = 2.1 Hz, 1H, H-2), 7.28 (d, *J*_H_ = 8.2 Hz, 1H, H-7), 7.32 (s, 1H, H-4), 10.99 (s, 1H, H-1). ^13^C NMR (DMSO-*d6*) δ: 13.8 (C-8), 18.2 (C-4′), 26.1 (C-5′), 34.6 (C-1′), 103.7 (C-3), 112.2 (C-7), 117.4 (C-4), 120.1 (C-9), 123.1 (C-6), 124.6 (C-2), 125.1 (C-2′), 126.7 (C-3a), 131.3 (C-3′), 132.4 (C-5), 135.3 (C-7a). The molecular formula was established as C_15_H_16_N_2_ by high resolution MS (*m*/*z* 225.1385 [M+H]^+^; calculated molecular weight for C_15_H_17_N_2_, 225.1386).

##### Expression and Purification of N-terminal His-tagged Proteins

A PCR fragment containing *SCO7467* and *SCO7468* was amplified from the genomic DNA of *S. coelicolor* A3(2) using the Expand High Fidelity PCR system and the primers listed in Table 1. After the *E. coli* BL21(DE3) that harbored pHIS8_SCO7467 or pHIS8_SCO7468 was cultured overnight in LB-kanamycin, then Terrific broth containing 50 μg ml^−1^ kanamycin was inoculated with the resultant cells and cultured at 37 °C. After a 2-h culture, isopropyl 1-thio-β-d-galactopyranoside was added to a final concentration of 0.5 mm. After overnight culture at 18 °C, cells were harvested by centrifugation and frozen at −80 °C. All subsequent steps were performed at 4 °C. After being thawed on ice, cells were suspended in a lysis buffer (50 mm Tris-HCl, pH 8.0, 500 mm NaCl, 20 mm imidazole, 20% (w/v) glycerol, and 1% Tween 20). The cell suspension was sonicated with a Branson Sonifier 250 (Emerson Japan, Tokyo, Japan). To separate the cellular debris from the soluble protein, the lysate was centrifuged at 17,000 rpm at 4 °C for 20 min. The supernatant was applied to a Ni-nitrilotriacetic acid column (Qiagen, Tokyo, Japan) that was equilibrated with the lysis buffer and washed with a buffer containing 50 mm Tris-HCl, pH 8.0, 500 mm NaCl, 20 mm imidazole, and 20% (w/v) glycerol. Then, recombinant protein was eluted using the wash buffer containing 250 mm imidazole.

To investigate the multimeric status of the recombinant proteins, a gel filtration analysis was performed. Before analysis, the column was equilibrated with a buffer containing 50 mm Tris-HCl, pH 8.0, and 150 mm NaCl, and SCO7467 was then applied to a Superdex 75 column (GE Healthcare), which was calibrated with conalbumin (75 kDa), ovalbumin (43 kDa), carbonic anhydrase (29 kDa), and ribonuclease A (13.7 kDa). In the case of SCO7468, a Superdex 200 column (GE Healthcare) was used. Before analysis, the column was equilibrated with a buffer containing 20 mm Tris-HCl, pH 8.0, and 150 mm NaCl, and SCO7468 was then applied to the equilibrated column, which was calibrated with aldolase (158 kDa), conalbumin, ovalbumin, carbonic anhydrase, and ribonuclease A.

##### Prenyltransferase Assay of SCO7467

The SCO7467 assay was performed in 100 mm Tris-HCl, pH 8.0, containing 2 mm MgCl_2_, 1 mm tryptophan or IAN, 1 mm DMAPP, and 1 mg ml^−1^ SCO7467. The reaction mixture was incubated at 30 °C for 10 min. After incubation, the reaction was quenched by the addition of an equal volume of methanol and mixed by vortexing. The mixture was centrifuged at 15,000 rpm for 1 min to remove protein. The supernatant was then subjected to LC-MS/MS analysis under the same conditions as described above. The large scale preparation of the SCO7467-catalyzed reaction product was performed in a total volume of 50 ml with tryptophan as a substrate. The reaction was performed in 100 mm Tris-HCl, pH 8.0, 1 mm tryptophan, 1 mm DMAPP, and up to 1 mg ml^−1^ SCO7467, which was added in two consecutive steps over 4 h. The reaction mixture was incubated at 30 °C for 16 h, and the reaction was quenched by the addition of 50 ml of methanol. After the precipitant was removed by filtration, all solvents were evaporated *in vacuo*, and the residue was dissolved in 1 ml of methanol. The enzymatic reaction product was purified by preparative HPLC with a PEGASIL ODS column (20 × 250 mm; Senshu Scientific) and isocratic elution of 60% MeOH + 0.1% acetic acid at a flow rate of 7.0 ml min^−1^; the column eluate was monitored at 276 nm.

##### NMR and MS Spectral Data of 5-DMAT

^1^H NMR (DMSO-*d6*) δ: 1.66 (s, 3H, H-5′), 1.68 (s, 3H, H-4′), 2.84 (dd, *J*_H_ = 9.6, 15.1 Hz, 1H, H-8), 3.27 (dd, *J*_H_ = 3.4, 15.1 Hz, 1H, H-8), 3.33 (d, *J*_H_ = 7.6 Hz, 2H, H-1′), 3.36 (dd, *J*_H_ = 3.4, 9.6 Hz, 1H, H-9) 5.29 (t, *J*_H_ = 7.6 Hz, 1H, H-2′), 6.85 (dd, *J*_H_ = 1.4, 8.3 Hz, 1H, H-6), 7.12 (s, 1H, H-2) 7.21 (d, *J*_H_ = 8.3 Hz, 1H, H-7), 7.28 (s, 1H, H-4), 10.74 (s, 1H, H-1). ^13^C NMR (DMSO-*d6*) δ: 18.2 (C-4′), 26.1 (C-5′), 27.8 (C-8), 34.6 (C-1′), 55.3 (C-9), 109.9 (C-3), 111.8 (C-7), 117.7 (C-4), 122.4 (C-6), 124.6 (C-2), 125.4 (C-2′), 127.9 (C-3a), 130.9 (C-3′), 131.6 (C-5), 135.4 (C-7a), 170.3 (C-10). The molecular formula was established as C_16_H_20_N_2_O_2_ by high resolution MS (*m/z* 273.1600 [M+H]^+^; calculated molecular weight for C_16_H_21_N_2_O_2_, 273.1598).

The large scale preparation of the SCO7467-catalyzed reaction product was performed in a total volume of 10 ml with IAN as the substrate. The reaction was performed in 100 mm Tris-HCl, pH 8.0, containing 2 mm MgCl_2_, 2 mm IAN, 2 mm DMAPP, and up to 1 mg ml^−1^ SCO7467, which was added in two consecutive steps over 4 h. The reaction mixture was incubated at 30 °C for 16 h and then extracted three times with 10 ml of ethyl acetate. The organic layer was evaporated *in vacuo*, and the residue was dissolved in 1 ml of methanol. The enzymatic reaction product was purified by preparative HPLC with a PEGASIL ODS column (20 × 250 mm; Senshu Scientific) and isocratic elution of 65% MeOH + 0.1% acetic acid at a flow rate of 7.0 ml min^−1^; the column eluate was monitored at 223 nm.

##### NMR and MS Spectral Data of 2-DMAIAN

^1^H NMR (DMSO-*d6*) δ: 1.67 (s, 3H, H-5′), 1.71 (s, 3H, H-4′), 3.44 (d, *J*_H_ = 6.8 Hz, 2H, H-1′), 3.93 (s, 2H, H-8), 5.26 (t, *J*_H_ = 6.8 Hz, 1H, H-2′), 6.97 (dd, *J*_H_ = 7.6, 7.6, Hz, 1H, H-5), 7.02 (dd, *J*_H_ = 7.6, 7.6 Hz, 1H, H-6), 7.27 (d, *J*_H_ = 7.6 Hz, 1H, H-7), 7.47 (d, *J*_H_ = 7.6 Hz, 1H, H-4), 10.9 (s, 1H, H-1). ^13^C NMR (DMSO-*d6*) δ: 12.5 (C-8), 18.3 (C-4′), 25.1 (C-1′), 26.0 (C-5′), 99.3 (C-3), 111.5 (C-7), 117.8 (C-4), 119.4 (C-5), 119.9 (C-9), 121.1 (C-2′), 121.4 (C-6), 127.6 (C-3a), 133.3 (C-3′), 135.7 (C-7a), 137.1 (C-2) The molecular formula was established as C_15_H_16_N_2_ by HRESI-MS (*m*/*z* 225.1382 [M+H]^+^; calculated molecular weight for C_15_H_17_N_2_, 225.1386).

##### NMR and MS Spectral Data of 6-DMAIAN

^1^H NMR (DMSO-*d6*) δ: 1.68 (s, 3H, H-4′), 1.68 (s, 3H, H-5′), 3.35 (d, *J*_H_ = 7.6 Hz, 2H, H-1′), 3.96 (s, 2H, H-8), 5.30 (t, *J*_H_ = 7.6 Hz, 1H, H-2′), 6.85 (d, *J*_H_ = 8.2 Hz, 1H, H-5), 7.12 (s, 1H, H-7), 7.22 (brs, 1H, H-2), 7.43 (d, *J*_H_ = 8.2 Hz, 1H, H-4), 10.92 (s, 1H, H-1) ^13^C NMR (DMSO-*d6*) δ: 13.8 (C-8), 18.2 (C-4′), 26.1 (C-5′), 34.5 (C-1′), 104.0 (C-3), 111.3 (C-7), 118.4 (C-4), 120.0 (C-9), 120.6 (C-2′), 124.0 (C-2), 124.7 (C-2′), 124.7 (C-3a), 131.6 (C-3′), 135.5 (C-5), 137.2 (C-7a) The molecular formula was established as C_15_H_16_N_2_ by HRESI-MS (*m*/*z* 225.1386 [M+H]^+^; calculated molecular weight for C_15_H_17_N_2_, 225.1386).

##### Steady-state Kinetics Study of SCO7467

A spectrophotometric SCO7467 assay using a coupled system with a pyrophosphate reagent (Sigma-Aldrich) was used to study the steady-state kinetics of SCO7467 because the SCO7467 prenyltransferase forms a pyrophosphate anion co-product during catalysis. This assay was essentially performed as described previously (16). Prenyltransferase activity was assayed in 100 mm Tris-HCl, pH 8.0, containing 2 mm MgCl_2_, l-tryptophan, DMAPP, and 267 μl of the pyrophosphate reagent in a total volume of 800 μl. When the concentration of DMAPP was fixed at 1 mm, the concentrations of tryptophan were varied at 15, 30, 60, 120, and 180 μm. When the concentration of tryptophan was fixed at 1 mm, the concentrations of DMAPP were varied at 10, 20, 40, 80, and 160 μm. After the reaction mixture containing no enzyme was incubated at 30 °C for 5 min, the reaction was started by adding 67 μg of SCO7467. The SCO7467-dependent oxidation of NADH was monitored using a UV-1600PC spectrophotometer (Shimadzu) equipped with a cell holder CPS-240A (Shimadzu) and maintained at 30 °C. Initial velocities were determined from the slope of a plot of NADH consumption *versus* incubation time. The molar extinction coefficient (ϵ) of NADH at 340 nm was 6220 m^−1^ cm^−1^. Steady-state kinetic parameters were calculated using the SigmaPlot 10.0 and Enzyme Kinetics Module 1.3 software (Systat Software, Point Richmond, CA).

##### The Enzymatic Activity of SCO7468

The SCO7468 assays were performed in 100 mm Tris-HCl, pH 8.0, containing 50 μm 5-DMAT or tryptophan, 1 mg ml^−1^ SCO7468, and 1 mm NADPH or NADH. The reaction mixtures were incubated at 30 °C for 10 min. After the incubation, the reaction was quenched by the addition of an equal volume of methanol and mixing by vortexing. The mixture was centrifuged at 15,000 rpm for 1 min to remove protein. The supernatant was subjected to LC-MS/MS analysis under the same conditions as described above. The large scale preparation of the SCO7468-catalyzed reaction product was performed in a total volume of 10 ml. The reaction was performed in 100 mm Tris-HCl, pH 8.0, containing 1 mm 5-DMAT, 1 mm NADPH, and up to 1 mg ml^−1^ SCO7468, which was added in two consecutive steps over 4 h. The reaction mixture was incubated at 30 °C for 16 h and then extracted twice with 10 ml of ethyl acetate. After drying over Na_2_SO_4_, the organic layer was evaporated *in vacuo*, and the residue was dissolved in 1 ml of methanol. The enzymatic reaction product was purified by preparative HPLC with a PEGASIL ODS column (20 × 250 mm; Senshu Scientific) and isocratic elution of 80% MeOH at a flow rate of 8.0 ml min^−1^; the column eluate was monitored at 275 nm.

##### NMR and MS Spectral Data of 5-DMAI-3-acetaldoxime (5-DMAIAOx)

^1^H NMR (CDCl_3_) δ: 1.74 (s, 3H, H-5′), 1.75 (s, 3H, H-4′), 3.48 (d, *J*_H_ = 7.1 Hz, 2H, H-1′), 3.64 (d, *J*_H_ = 6.2 Hz, 2H, H-8 (*anti*)), 3.83 (d, *J*_H_ = 5.0 Hz, 2H, H-8 (*syn*)), 5.38 (t, *J*_H_ = 7.1 Hz, 1H, H-2′), 6.94 (t, *J*_H_ = 5.0 Hz, 1H, H-9 (*syn*)), 7.01 (brs, 1H, H-2 (*anti*)), 7.04 (brs, 1H, H-2 (*syn*)), 7.04 (m, 1H, H-6*), 7.05 (m, 1H, H-6*), 7.27 (d, *J*_H_ = 7.2 Hz, 1H, H-7*), 7.29 (d, *J*_H_ = 7.7 Hz, 1H, H-7*), 7.37 (brs, 1H, H-4*), 7.38 (brs, 1H, H-4*), 7.60 (t, *J*_H_ = 6.2 Hz, 1H, H-9 (*anti*)), 7.95 (s, 1H, H-1). ^13^C NMR (CDCl_3_) δ: 18.0 (C-4′), 21.6 (C-8 (*syn*)), 25.9 (C-5′), 26.0 (C-8 (*anti*)), 34.6 (C-1′), 110.3 (C-3 (*anti*)), 110.7 (C-3 (*syn*)), 111.2 (C-7), 117.7 (C-4*), 117.8 (C-4*), 122.4 (C-6*), 122.5 (C-6*), 123.4 (C-2), 124.5 (C-2′), 127.5 (C-3a), 131.8 (C-3′), 133.3 (C-5), 134.9 (C-7a), 151.0 (C-9 (*anti*)), 151.9 (C-9 (*syn*)). (Asterisks indicate that we were unable to assign *syn* or *anti* for these peaks.) The molecular formula was established as C_15_H_18_N_2_O by high resolution MS (*m*/*z* 243.1495 [M+H]^+^; calculated molecular weight for C_15_H_19_N_2_O, 243.1492).

##### Identification of N-Hydroxytryptophan as a Reaction Intermediate of SCO7468

*N*-Hydroxytryptophan was synthesized by reductive amination of indole-3-pyruvic acid. Indole-3-pyruvic acid oxime, which was derived from indole-3-pyruvic acid and hydroxylammonium chloride, was reduced by 2-picoline-borane according to the method described in the literature (17, 18). The SCO7468 assay with *N*-hydroxytryptophan was performed in 100 mm Tris-HCl, pH 8.0, containing 1 mm *N*-hydroxytryptophan, 1 mg ml^−1^ SCO7468, and 1 mm NADPH. The reaction mixture was incubated at 30 °C for 60 min. After the incubation, the reaction was quenched by the addition of an equal volume of methanol and mixed by vortexing. The resulting mixture was centrifuged at 15,000 rpm for 1 min to remove protein. The supernatant was subjected to LC-MS/MS analysis under the same conditions as described above.

##### Steady-state Kinetics Study of SCO7468

SCO7468 kinetic assays were performed in a reaction mixture containing 100 mm Tris-HCl, pH 8.0, 20 μm FAD, and 1 mm NADPH. The reactions with 5-DMAT were carried out in 0.2 ml of buffer, and the reactions with tryptophan were carried out in 0.1 ml of buffer. The concentrations of 5-DMAT were 62.5, 125, 250, 500, and 1000 nm, whereas the concentrations of tryptophan were 0.625, 1.25, 2.5, 5.0, and 10 mm. After the reaction mixture containing no enzyme was incubated at 30 °C for 1 min, the reaction was started by adding SCO7468 at a final concentration of 0.1 mg ml^−1^. After the 40-s reaction with 5-DMAT, the reaction mixture was extracted three times by an equal amount of ethyl acetate and dried *in vacuo*. The dried residues were dissolved in 40 μl of methanol and analyzed by LC-MS/MS. The 8-min reactions with 0.625, 1.25, and 2.5 mm l-tryptophan and the 2-min reactions with 5.0 and 10 mm l-tryptophan were quenched by the addition of an equal volume of methanol. The resultant methanol solutions were analyzed directly using an LC-MS/MS system equipped with a CAPCELL PAK C_18_ IF column (2.0 × 50 mm; column temperature, 40 °C) under the following conditions: mobile phase A, water + 0.1% formate; mobile phase B, acetonitrile + 0.1% formate; 50% B for 5 min at a flow rate of 0.4 ml min^−1^. The amount of the product was calculated from a standard curve, which was obtained from LC-MS/MS fragmentation analysis using the multiple reaction monitoring mode. The peak area of a characteristic product ion resulting from each precursor ion corresponding to 5-DMAIAOx (*m*/*z* 243.1) or IAOx (*m*/*z* 175.1) was used for quantification; a characteristic product ion (*m*/*z* 187.1) at 1.38 min was selected for 5-DMAIAOx, and a characteristic product ion (*m*/*z* 158.1) at 0.61 min was selected for IAOx.

## RESULTS

### 

#### 

##### Indole Prenyltransferase Genes Are Widely Distributed in Actinomycetes

Takahashi *et al.* (10) reported the presence of IptA indole prenyltransferase homologs in *S. coelicolor* A3(2) and *Streptomyces ambofaciens* ATCC23877, but no mention has been made of the distribution of IptA indole prenyltransferase homologs in other actinomycetes. Therefore, to determine the distribution of IptA-containing gene clusters in other actinomycetes, we performed a BLAST search against the National Center for Biotechnology Information (NCBI) database using an amino acid sequence of IptA as a query. Surprisingly, the database searches retrieved >10 IptA homologs in various actinomycetes (Fig. 1). In addition, the gene organizations harboring the indole prenyltransferase gene were very similar to each other. All of these gene clusters had a four-gene cassette including the sensor-like histidine kinase gene, two hypothetical genes, and an ATP/GTP-binding protein gene. This gene cassette is known as a “conservon,” which is a gene cassette conserved among actinomycetes (4, 19–21), some of which have an additional cytochrome P-450 gene. The database search results allowed us to classify these gene clusters into two types (here, we designate them Type A and Type B) based on differences in their constituent genes; the Type A gene cluster includes a tryptophanase gene, whereas the Type B gene cluster includes a FMO gene (Fig. 1). Although Takahashi *et al.* have demonstrated that one of the Type A gene clusters is responsible for the biosynthesis of 6-DMAI-3-carbaldehyde, no functions of the Type B gene cluster have been elucidated to date.

##### Heterologous Expression of the Prenyltransferase Gene and the FMO Gene

To identify the metabolite originating from the expression of the *S. coelicolor* A3(2) Type B gene cluster containing *SCO7467* and *SCO7468*, we performed heterologous expression of *SCO7467* and *SCO7468* using *S. lividans* TK23 as a host strain. Because *S. lividans* TK23 possesses a gene cluster identical to that of *S. coelicolor* A3(2), we expected a gene dosage effect on the production of an unidentified metabolite caused by the introduction of *SCO7467* and *SCO7468* into *S. lividans* TK23.

**FIGURE 2.**
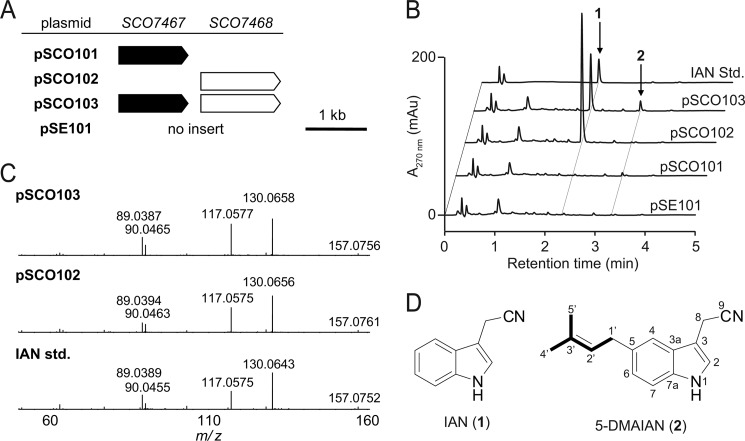
**Heterologous expression of *SCO7467* and *SCO7468* in *S. lividans* TK23.** *A*, plasmids used for heterologous expression. *B*, HPLC analysis of the metabolites of the *S. lividans* TK23 transformants harboring pSE101, pSCO101, pSCO102, or pSCO103. *S. lividans* TK23/pSE101 was used as a control. *C*, LC-MS/MS fragmentation patterns of authentic IAN and metabolites from *S. lividans* TK23/pSCO103 and *S. lividans* TK23/pSCO102. *D*, structures of IAN (**1**) and 5-DMAIAN (**2**).

For heterologous expression, the plasmid pSCO101 (containing *SCO7467*), pSCO102 (containing *SCO7468*), or pSCO103 (containing *SCO7467* and *SCO7468*) was individually introduced into *S. lividans* TK23 (Fig. 2*A*). As a control strain, *S. lividans* TK23 harboring the empty vector pSE101 was used. Introduction of *SCO7467* did not cause apparent changes in the metabolic profile, whereas introduction of *SCO7468* caused the production of an unidentified product that showed an indole-like UV spectrum. LC-MS/MS analysis unambiguously identified this product as IAN (**1**) (Fig. 2, *C* and *D*). A transformant that harbored both *SCO7467* and *SCO7468* also produced **1**. In addition to **1**, this transformant produced unidentified product **2**, which showed a UV spectrum similar to **1** but with a longer chromatographic retention time than that of **1** (Fig. 2*B*). Although this product **2** was also produced at a negligible level by *S. lividans* TK23 transformants harboring the empty vector or *SCO7467* alone, introduction of both *SCO7467* and *SCO7468* increased the production of the unidentified IAN-like product. The large scale preparation of **2** allowed us to deduce its structure to be 5-DMAIAN (**2**) (Fig. 2*D*) on the basis of NMR and high resolution MS spectral data.

SCO7467 displayed 59% amino acid sequence identity to IptA, which catalyzes a dimethylallyl group transfer to the C-6 position of tryptophan (**3**). In addition, the introduction of SCO7467 into *S. lividans* TK23 increased the production of **2** (IAN with a dimethylallyl group at C-5) in this transformant. Both facts suggest that SCO7467 is involved in the attachment of a dimethylallyl group into an indole derivative. Taken together, these data suggest that SCO7467 appends a dimethylallyl group to the C-5 of **3** to yield 5-DMAT (**4**). Next, SCO7468 presumably uses **4** as a substrate in the formation of **2**. To verify this hypothesis, we next performed *in vitro* analysis with the recombinant SCO7467 and SCO7468 prepared from the *E. coli* transformants.

##### Expression and Purification of Recombinant SCO7467 and SCO7468

**FIGURE 3.**
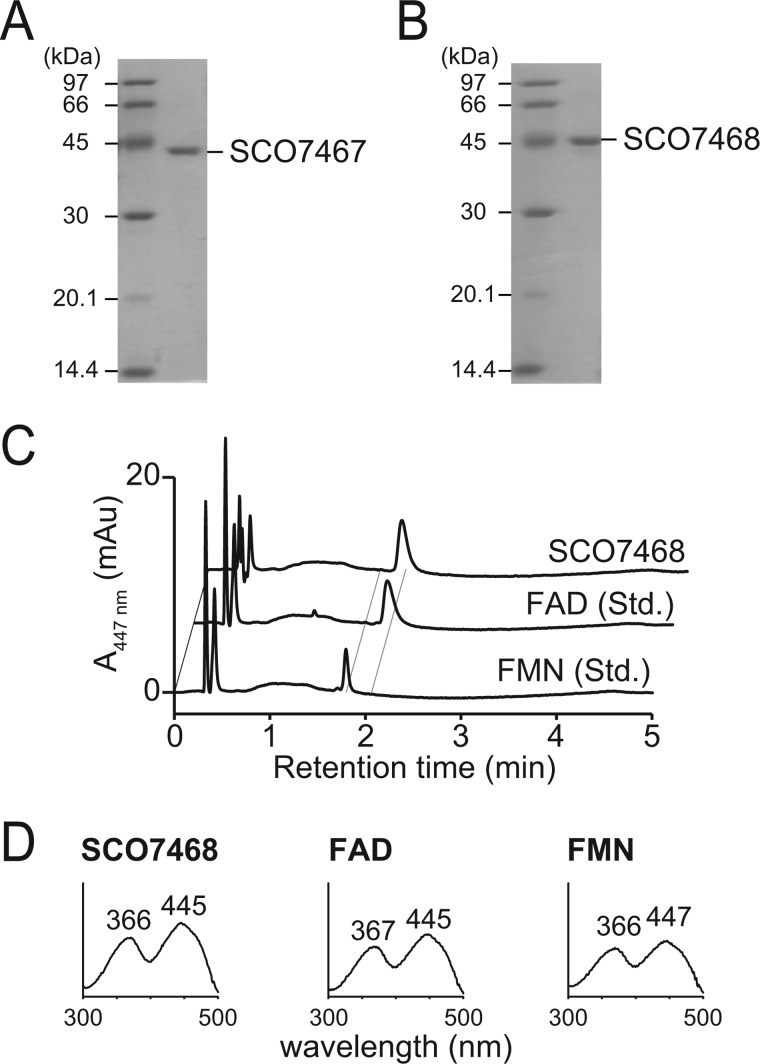
**Characterization of recombinant SCO7467 and SCO7468.** *A*, SDS-PAGE of the recombinant SCO7467. *B*, SDS-PAGE of the recombinant SCO7468. *C*, HPLC analysis of SCO7468-bound flavin cofactor. *D*, UV-visible spectra of flavin co-factors in each sample.

The N-terminal His_8_-tagged SCO7467 and SCO7468 were overexpressed in *E. coli*, and the recombinant proteins were purified to homogeneity by a Ni-nitrilotriacetic acid column. The molecular mass of SCO7467 was estimated to be 40 kDa by SDS-PAGE and gel filtration chromatography, suggesting that SCO7467 is likely a monomer (Fig. 3*A*). The molecular mass of SCO7468 was estimated to be 45 kDa by SDS-PAGE and 90 kDa by gel filtration chromatography, suggesting that SCO7468 is likely a dimer (Fig. 3*B*). Purified SCO7468 was yellow, suggesting that the enzyme contained a flavin co-factor and was successfully expressed as an active enzyme. Denaturation of SCO7468 with methanol followed by reverse-phase HPLC analysis showed a major peak identical to FAD, indicating that FAD existed in the recombinant SCO7468 protein as a tightly bound form (Fig. 3*C*).

##### In Vitro Analysis of Recombinant SCO7467

**FIGURE 4.**
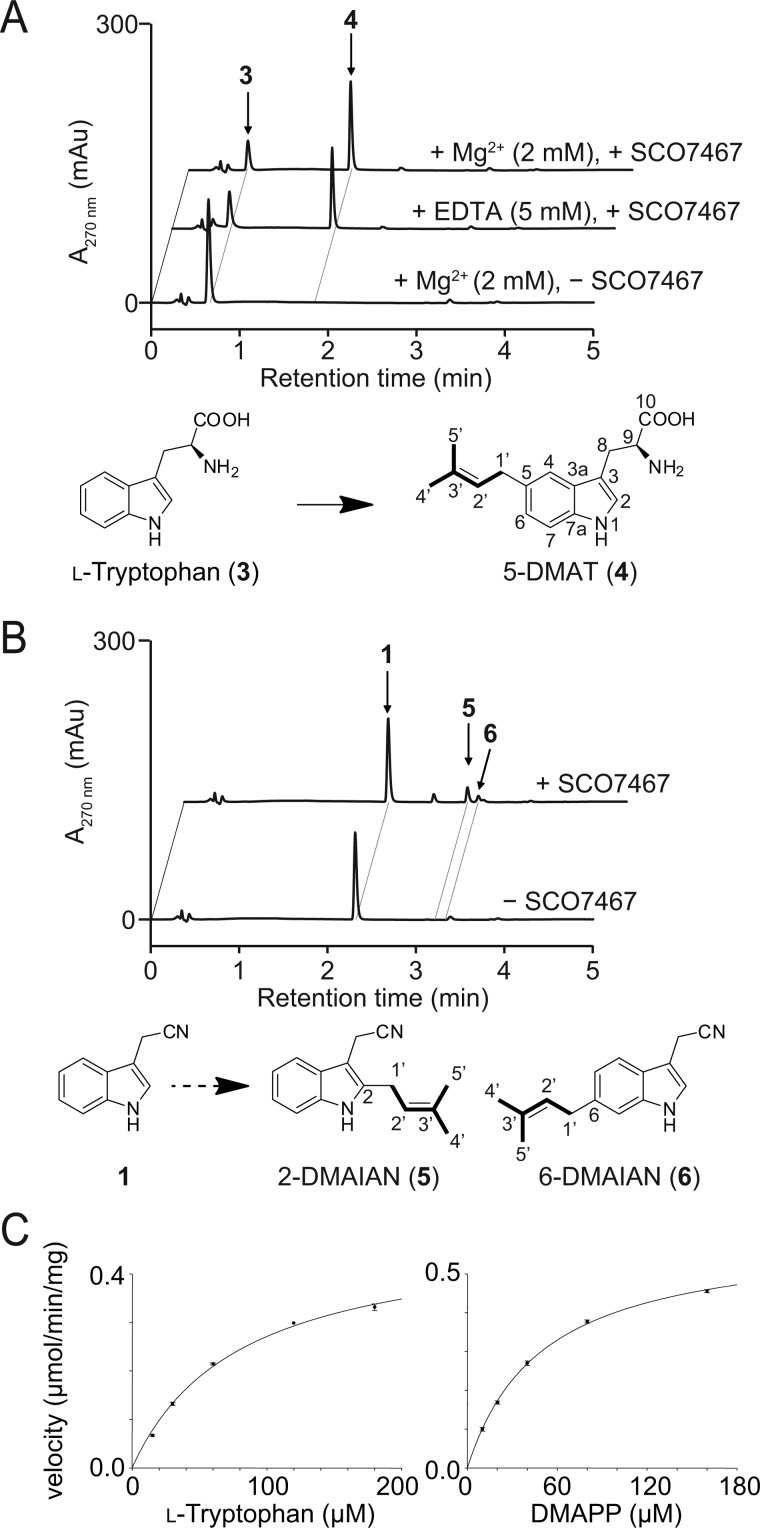
**Prenyltransferase activity of recombinant SCO7467.** *A*, *in vitro* analysis of the SCO7467 reaction with l-tryptophan (**3**). SCO7467 efficiently converted **3** to 5-DMAT (**4**) over a 10-min incubation. *B*, *in vitro* analysis of the SCO7467 reaction with **1**. SCO7467 catalyzed the prenylation of **1** to give a small amount of 2-DMAIAN (**5**) and 6-DMAIAN (**6**). *C*, steady-state kinetics of SCO7467. Michaelis-Menten plots of the SCO7467-catalyzed reactions using various concentrations of l-tryptophan (*left*) and DMAPP (*right*) are shown. The concentration of DMAPP or l-tryptophan was fixed at 1 mm when the concentration of the other substrate varied.

SCO7467 showed 59% amino acid sequence identity to l-tryptophan:dimethyltransferase IptA, which appends a dimethylallyl group at the C-6 position of **3** to form 6-DMAT. This high amount of shared identity led us to expect that SCO7467 would accept **3** as a substrate. To assess enzyme activity, we incubated SCO7467 with **3** in the presence of DMAPP. The formation of one product with a dimethylallyl group was readily detected by HPLC (Fig. 4*A*). Large scale incubation of SCO7467 with **3** and DMAPP produced a sufficient amount of the product to permit its structural elucidation using both MS and NMR analyses. The product possessed a single dimethylallyl chain at C-5 and was identified as 5-DMAT (**4**). This SCO7467-catalyzed reaction indicates that SCO7467 is capable of converting **3** to **4**.

Similar to IptA, SCO7467 also catalyzed the prenylation of **3** in the absence of Mg^2+^ and maintained its activity even in the presence of 5 mm EDTA, indicating that the SCO7467-catalyzed prenylation reaction is Mg^2+^-independent (Fig. 4*A*). We then performed steady-state kinetic analysis, which demonstrated that the SCO7467 reaction followed Michaelis-Menten kinetics. The apparent *K_m_* values were 80 ± 6 μm for **3** and 50 ± 2 μm for DMAPP at fixed saturating concentrations of DMAPP (1 mm) and **3** (1 mm), respectively. The turnover number *k*_cat_ of the reaction was 0.40 ± 0.1 s^−1^ (Fig. 4*C*). Meanwhile, the *in vivo* results mentioned above also suggested the possibility that SCO7467 appends a dimethylallyl group to the C-5 of **1** to give **2**. To verify this possibility, we incubated SCO7467 with **1** in the presence of DMAPP. This reaction resulted in the formation of several products (Fig. 4*B*). We isolated two of the products and determined the structure of each compound by NMR and HRESI-MS. The structure of **5** was determined to be 2-dimethylallylindole-3-acetonitrile (2-DMAIAN, **5**), whereas **6** was determined to be 6-dimethylallylindole-3-acetonitrile (6-DMAIAN, **6**) (Fig. 4B). This *in vitro* result was obviously inconsistent with the finding that no other indole compounds, with the exception of **1** and **2**, were produced in the heterologous expression experiment. We thus excluded the possibility that SCO7467 appends a dimethylallyl group to the C-5 of **1** to give **2** and concluded that *SCO7467* encodes a 5-dimethylallyltryptotphan synthase (l-tryptotphan:5-dimethylallyltransferase).

##### In Vitro Analysis of Recombinant SCO7468

**FIGURE 5.**
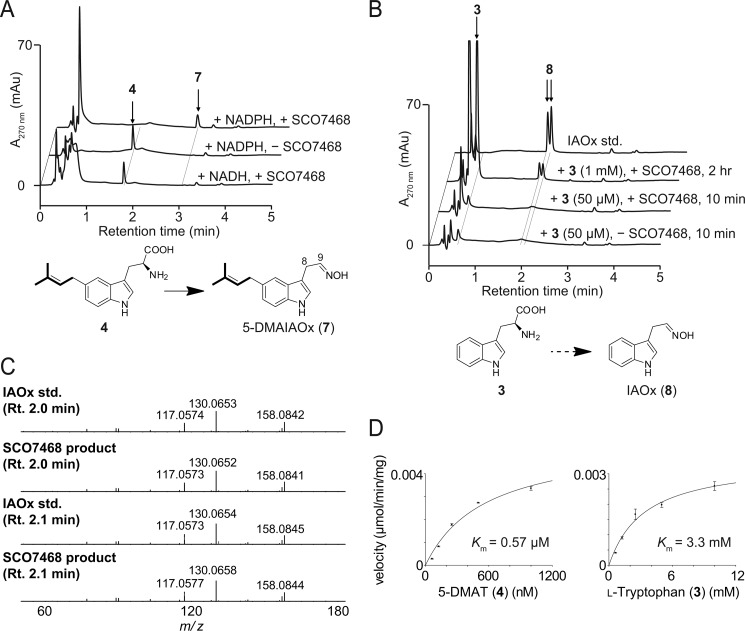
***In vitro* analysis of the SCO7468 reaction.** *A*, *in vitro* analysis of the SCO7468 reaction with 5-DMAT (**4**). SCO7468 completely converted **4** to 5-DMAIAOx (**7**) in an NADPH-dependent manner over a 10-min incubation. *B*, *in vitro* analysis of the SCO7468 reaction with **3**. The 10-min incubation of SCO7468 resulted in almost no substrate depletion and almost no product formation. In contrast, the prolonged 2-h incubation with 1 mm **3** resulted in the formation of IAOx (**8**). *C*, LC-MS/MS fragment patterns of authentic IAOx and SCO7468 reaction products detected in the chromatograms *B. D*, steady-state kinetics of SCO7468. Michaelis-Menten plots of the SCO7468-catalyzed reactions using various concentrations of 5-DMAT (*left*) and l-tryptophan (*right*) are shown. See “Experimental Procedures” for details.

The bioinformatic analyses of SCO7468 revealed that SCO7468 had 34% amino acid sequence identity to dimethylaniline monooxygenase (*N*-oxide-forming) (accession number EHA99148), suggesting that this enzyme is involved in the formation of an *N*-oxide from an amine. To assess enzyme activity, **4** was used as a substrate in the SCO7468 reaction because SCO7467 catalyzes the prenylation of **3** to give **4**. HPLC analysis of the reaction mixture readily revealed the enzyme-dependent formation of an unknown product **7** (Fig. 5A). After a 10-min incubation, SCO7468 converted all of **4** (50 μm) to **7**. The enzymatic activity was strictly dependent on β-NADPH; no activity was detected in the presence of β-NADH. Large scale incubation of SCO7468 with **4** and DMAPP produced a sufficient amount of **7** to permit its structural elucidation using both MS and NMR analyses. The structure was determined to be a mixture of *syn*- and *anti*-isomers of 5-DMAIAOx (**7**).

Meanwhile, **1** accumulated when SCO7468 was expressed in *S. lividans* (Fig. 2*B*), which suggests that SCO7468 is involved in the formation of **1** *in vivo*. Therefore, to further verify the function of SCO7468, we incubated SCO7468 with **3** in the presence of β-NADPH. However, the 10-min incubation of SCO7468 with 50 μm **3** resulted in almost no substrate depletion and almost no product formation (Fig. 4*B*). In contrast, the prolonged incubation (2 h) of SCO7468 with a higher concentration of **3** (1 mm) resulted in the clear formation of IAOx (**8**) (Fig. 5, *B* and *C*).

**FIGURE 6.**
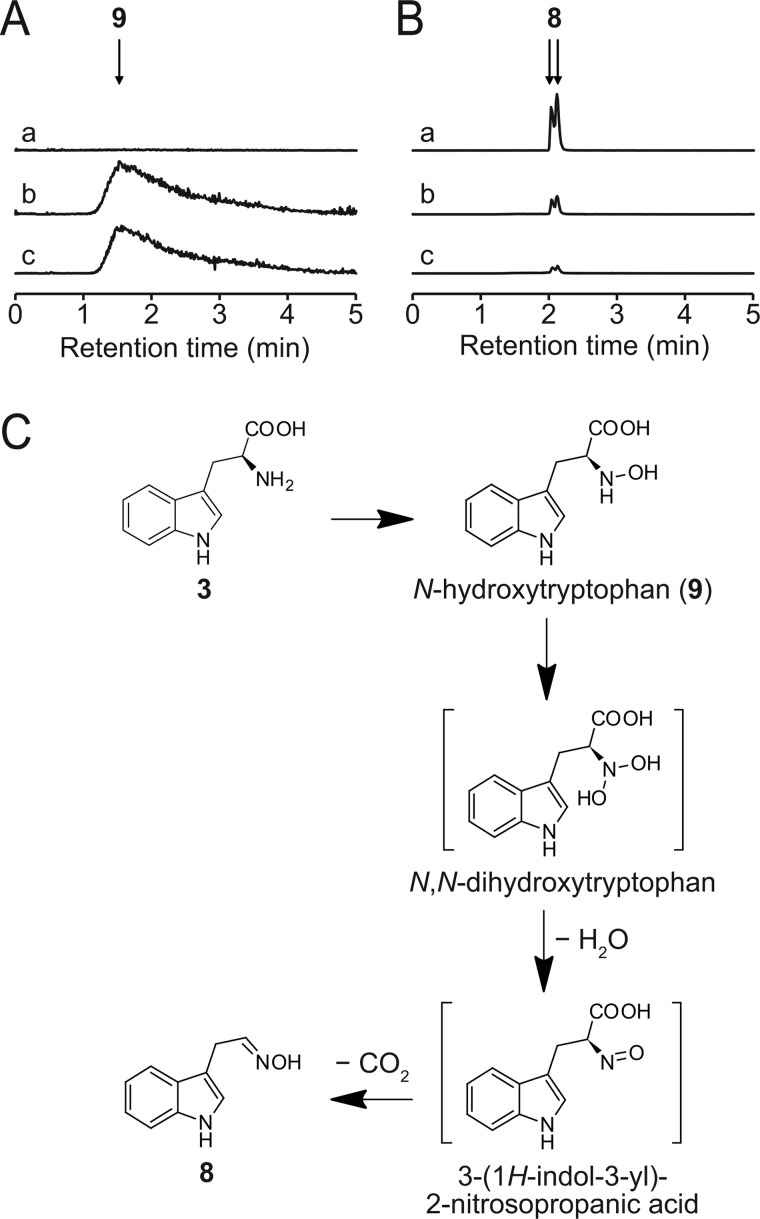
***In vitro* analysis of the SCO7468 reaction using *N*-hydroxytryptophan as a substrate.** *A*, extracted ion current chromatograms at *m*/*z* 221.0921 ± 0.0005 for *N*-hydroxytryptophan (**9**). Reaction mixtures: *a*, with SCO7468 for 60 min; *b*, without SCO7468 for 60 min; *c*, 0 min (control). *N*-Hydroxytryptophan (**9**) was completely consumed in a 60-min incubation with SCO7468 (*a*). *B*, extracted ion current chromatograms at *m*/*z* 175.0866 ± 0.0005 for IAOx (**8**). Reaction mixtures: *a*, with SCO7468 for 60 min; *b*, without SCO7468 for 60 min; *c*, 0 min (control). IAOx (**8**) was fully produced in a 60-min incubation with SCO7468 (*a*). Nonenzymatic formation of **8** was also detected at a trace level (*b* and *c*). *C*, proposed reaction mechanism for the SCO7468-catalyzed formation of **8** from **3** via **9**. *N*,*N*-Dihydroxytryptophan and 3-(1*H*-indol-3-yl)-2-nitrosopropanic acid are deduced intermediates of the SCO7468 reaction.

The SCO7468-catalyzed formation of IAOx (**8**) from tryptophan (**3**) is similar to that of *p*-hydroxyphenylacetaldoxime from l-tyrosine in cyanogenic glucoside biosynthesis, where cytochrome P-450_TYR_ likely catalyzes two successive *N*-hydroxylations of l-tyrosine followed by nonenzymatic decarboxylation to form *p*-hydroxyphenylacetaldoxime (22). To investigate whether SCO7468 catalyzes the aldoxime formation through the same reaction mechanism as that of cytochrome P-450_TYR_, we synthesized *N*-hydroxytryptophan (**9**), a possible intermediate of the SCO7468 reaction, and evaluated whether **9** could be converted into **8**. *N*-Hydroxytryptophan (**9**) was incubated with SCO7468 in the presence of NADPH. The incubation revealed that **8** was produced concomitantly with the complete consumption of **9** (Fig. 6), which unambiguously demonstrates that **9** is a reaction intermediate of SCO7468.

We then determined the kinetic parameters of SCO7468 toward **4** and **3**. *K_m_* and *k*_cat_ values for **4** were estimated to be 0.57 ± 0.1 μm and (8.2 ± 0.7) ×10^−3^ s^−1^, respectively, whereas *K_m_* and *k*_cat_ values for **3** were estimated to be (3.3 ± 0.7) ×10^3^ μm and (5.2 ± 0.4) ×10^−3^ s^−1^, respectively (Fig. 5*D*). This notable difference clearly indicates that **4** is a physiological substrate of SCO7468.

## DISCUSSION

In this study, we demonstrated that *S. coelicolor* A3(2) and *S. lividans* TK23 have the ability to biosynthesize a novel natural product, **2**. Although similar gene clusters responsible for the biosynthesis of **2** are widely distributed in actinomycetes, this specific function has not yet been elucidated. The heterologous expression in *S. lividans* of the structural genes *SCO7467* and *SCO7468* enabled us to identify **2**. *S. lividans* harboring the empty vector alone also produced **2** but at a negligible level (Fig. 2). This observation indicates that the biosynthetic genes responsible for the production of **2** are expressed only at an extremely low level. Therefore, under normal culture conditions, **2** may not be detected in the culture broth of *S. lividans*. In contrast, an *S. lividans* transformant efficiently expressing both *SCO7467* and *SCO7468* produced **2** at an easily detectable level (Fig. 2). These results indicate that introduction of exogenous *SCO7467* and *SCO7468* into *S. lividans* effectively increases the production of **2**. The present study demonstrated that a simple strategy such as heterologous expression is widely applicable for the identification of unidentified secondary metabolites synthesized by uncharacterized gene clusters that are buried in the public genome database. Identification of such metabolites would contribute to our knowledge of the structural diversity of natural products.

We also elucidated the biochemical functions of SCO7467 and SCO7468 in the biosynthesis of **2**. SCO7467 catalyzes the initial reaction in the biosynthesis and transfers a dimethylallyl group to C-5 of an indole nucleus of **3**. Despite high sequence similarity between SCO7467 and IptA, their regiospecificities of prenylation are different. IptA transfers a dimethylallyl group to C-6 of an indole nucleus of **3**. Recently, Yu *et al.* identified a fungal 5-dimethylallyltryptophan synthase from *Aspergillus clavatus* (23), but SCO7467 is the first actinomycetes prenyltransferase known to catalyze the addition of a dimethylallyl group to C-5 of **3**. However, fungal 5-dimethylallyltryptophan synthase and SCO7467 exhibit a low level of overall sequence similarity, although they catalyze the same reaction. Crystallographic analysis would be necessary to understand the mechanism of the regiospecificity of these prenyltransferases.

After the prenylation catalyzed by SCO7467, the FMO enzyme SCO7468 readily catalyzes the conversion of **4** to **7**, as its *K_m_* value (0.57 μm) toward **4** is quite low. SCO7468 also accepts **3** as the substrate, although its *K_m_* value (3.3 × 10^3^ μm) toward **3** is much higher than that for **4**. In fact, prolonged incubation of SCO7468 with the higher concentration of **3** resulted in the formation of **8**, which might explain the significant accumulation of **3** in the culture broth of the *S. lividans* TK23 transformant harboring pSCO102. In addition, a low *in vivo* concentration of DMAPP, one of the two substrates of SCO7467, might also explain the significant accumulation of **1**. If the concentration of DMAPP is limited *in vivo*, the SCO7467-catalyzed dimethylallyl transfer to **3** only occurs to a minimum extent, allowing the SCO7468-catalyzed conversion of **3** to **8** to take precedence *in vivo*. Meanwhile, the notable difference of kinetic parameters described above clearly indicates that **4** is a physiological substrate of SCO7468. We thus conclude that the FMO enzyme SCO7468 catalyzes the conversion of **4** to **7** and is responsible for the second step in the biosynthesis of **2**. In the final step of the biosynthesis of **2**, an unidentified enzyme(s) such as a promiscuous dehydratase, presumably dehydrates both **7** and **8** to give **2** and **1**, respectively, both of which were detected as final products in the *S. lividans* transformant harboring both SCO7467 and SCO7468.

A precedent for the conversion of **3** to **8** has been demonstrated in the biosynthesis of the plant hormone auxin in *Arabidopsis thaliana*. However, in the plant, the conversion is catalyzed by the action of the cytochrome P-450 enzyme CYP79B2 (24, 25). The finding that actinomycetes and plants utilize different types of oxygenases for the formation of **8** from **3** led us to investigate the reaction mechanism. We then identified **9** as a reaction intermediate of the FMO enzyme SCO7468. This identification suggests that the aldoxime formation from l-tryptophan catalyzed by SCO7468 proceeds via *N*,*N*-dihydroxytryptophan (Fig. 6). Presumably, *N*,*N*-dihydroxytryptophan nonenzymatically dehydrates to yield 3-(1*H*-indol-3-yl)-2-nitrosopropanoic acid, which successively decarboxylates to produce **8**. SCO7468 likely converts **4** to **7** by the same reaction mechanism. A precedented reaction mechanism has been presumed in the formation of *p*-hydroxyphenylacetaldoxime from l-tyrosine catalyzed by cytochrome P-450_TYR_ (also known as CYP79A1) from the plant *Sorghum bicolor* (22). However, an aldoxime-forming reaction by a FMO has been identified only in the metabolism of primary amines (26, 27). Thus, SCO7468 represents an unprecedented FMO that catalyzes two successive *N*-hydroxylation reactions of tryptophan-related amino acids to form the corresponding aldoximes.

**FIGURE 7.**
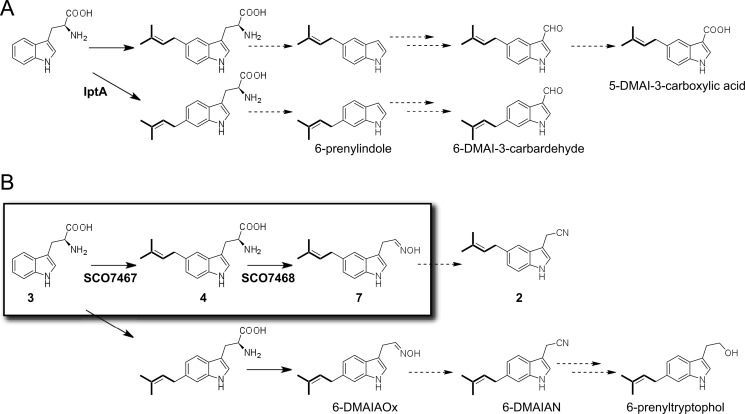
**Proposed pathway for prenylated indole biosynthesis.** *A*, l-tryptophan metabolism in actinomycetes by the Type A gene cluster. *B*, l-tryptophan metabolism in actinomycetes by the Type B gene cluster. The reactions demonstrated in this study are *framed*.

BLAST searches against the NCBI database revealed a wide distribution of the indole prenyltransferase-containing gene cluster among various actinomycetes. Some prenylated indole derivatives have also been isolated from *Streptomyces* species (10, 28–30). However, no studies on the biosynthesis of prenylated indoles have been reported other than the identification of an l-tryptophan:6-dimethylallyltransferase responsible for the biosynthesis of 6-DMAI-3-carbardehyde (10). The gene cluster involved in the biosynthesis of 6-DMAI-3-carbardehyde contains a tryptophanase. The presence of a tryptophanase gene in the cluster may explain the biosynthesis of 6-prenylindole isolated from *Streptomyces* sp. TP-A0595 (30). 6-Prenylindole is likely an intermediate in the biosynthesis of 6-DMAI-3-carbardehyde. This aldehyde moiety may be further oxidized to carboxylic acid as observed for the 5-DMAI-3-carboxylic acid in *Streptomyces* sp. MS239 (28). 6-DMAIAN has been isolated from the mycelium of *Streptomyces* sp. (BL-49-58-005) during a screening program for cytotoxic compounds (29). This strain produces 6-DMAIAOx and 6-prenyltryptophol as well as 6-DMAIAN. Despite the differences in their prenylated positions, **2** and 6-DMAIAN might be synthesized through similar pathways where indole prenyltransferase first appends a dimethylallyl group to **3** and FMO then catalyzes the conversion of DMAT into 5- or 6-DMAIAOx, followed by conversion into 5- or 6-DMAIAN by an unidentified dehydratase. *Streptomyces* sp. (BL-49-58-005) may have both a nitrilase and a reducing enzyme that act to metabolize 6-DMAIAN into 6-prenyltryptophol. Thus, the present study provides insight into the biosynthesis of prenylated indoles that have been purified from actinomycetes. The proposed biosynthetic pathway of the prenylated indoles is summarized in Fig. 7.

Prenylated indole derivatives are widely distributed in nature and show diverse biological and pharmacological activities including cytotoxicity and antifungal activity. In addition, because the potential for prenylated indole biosynthesis is widely distributed in soil actinomycetes, the prenylated indoles identified in the present study might possess yet unknown and important biological activities. Particularly intriguing is the structural similarity between **2** and indole-3-acetic acid (auxin), a major plant hormone. Recently, transkingdom signaling between *Pseudomonas aeruginosa* and *A. thaliana* was reported (31). *P. aeruginosa* produces diketopiperazine derivatives of cyclodepsipeptides that are involved in plant growth promotion. Considering that actinomycetes mainly live in soil, which is a rhizosphere of plants, prenylated indoles such as **2** might contribute to an uncharacterized signaling pathway between actinomycetes and plants. Such signaling might involve the conservon that is always located upstream of the structural biosynthetic genes of **2** and resembles the eukaryotic G protein-coupled regulatory system. Investigation of the biological activity of **2** against *A. thaliana* is currently underway in our laboratory.

## Footnotes

3 The abbreviations used are:

FMO: flavin-dependent monooxygenase
DMAI: dimethylallylindole
DMAIAN: dimethylallylindole-3-acetonitrile
5-DMAIAOx: 5-dimethylallylindole-3-acetaldoxime
DMAPP: dimethylallyl diphosphate
DMAT: dimethylallyltryptophan
DMSO: dimethyl sulfoxide
IAN: indole-3-acetonitrile
IAOx: indole-3-acetaldoxime
MS/MS: tandem MS
HRESI: high-resolution electrospray ionization.
